# Antenatal Betamethasone Exposure at the Threshold of Viability: Survival and Neonatal Outcomes at 21–25 Weeks of Gestation

**DOI:** 10.3390/children13101396

**Published:** 2026-10-14

**Authors:** Mehtap Durukan Tosun, Cuma Tasin, Nihan Ozel Ercel, Huseyin Simsek, Mustafa Akcalı, Huseyin Durukan

**Affiliations:** 1 Neonatology, Mersin City and Education Hospital, 33800 Mersin, Turkey; 2 Department of Obstetrics and Gynecology, Mersin City and Education Hospital, 33800 Mersin, Turkey; 3 Department of Biostatistics and Informatics in Medicine, Faculty of Medicine, Mersin University, 33110 Mersin, Turkey; 4 Department of Obstetrics and Gynecology, Faculty of Medicine, Mersin University, 33343 Mersin, Turkey

**Keywords:** immaturite, antenatal corticosteroids, betamethasone, neonatal mortality, neonatal morbidity, survival

## Abstract

**Highlights:**

**What are the main findings?**

• Antenatal betamethasone exposure was associated with higher survival and survival without severe morbidity in periviable infants.
• Complete antenatal betamethasone exposure was associated with lower rates of severe IVH or death, NEC or death, and ROP or death.

**What is the implication of the main finding?**

• These findings raise the question of whether the lower gestational-age threshold for routine antenatal corticosteroid exposure could be extended below 24 weeks in carefully selected periviable pregnancies.
• Further prospective and multicenter studies are needed to determine the potential benefits and risks of antenatal corticosteroid exposure before 24 weeks of gestation.

**Abstract:**

**Introduction:** Survival and short-term neonatal outcomes in preterm infants remain a significant clinical challenge. Although it is known that antenatal corticosteroids reduce neonatal mortality and morbidity when administered between 24 and 34 weeks of gestation, evidence regarding their use in borderline pregnancies is limited. The aim of this study was to evaluate the effect of antenatal betamethasone exposure on neonatal mortality and morbidity in infants born between 21 and 25 weeks of gestation. **Materials and Methods:** This retrospective study includes newborns born at Mersin City Training and Research Hospital between 1 January 2021 and 1 January 2026, at gestational ages ranging from 210/7 to 256/7 weeks, who underwent resuscitation in the delivery room and were admitted to the neonatal intensive care unit. Neonatal clinical and maternal characteristics, pregnancy and delivery characteristics, antenatal corticosteroid exposure, the need for respiratory support, neonatal morbidities, and mortality data were evaluated. The primary outcome was survival to discharge or transfer alive; secondary outcomes included recorded neonatal morbidities and their composites with death. Descriptive statistics were presented as medians (25th–75th percentiles), counts, and percentages. Group comparisons used the Kruskal–Wallis or Mann–Whitney U test for continuous variables and Fisher exact or Fisher–Freeman–Halton exact tests for binary categorical variables. Adjusted logistic regression assessed associations with clinical outcomes; sensitivity analyses included restriction to singleton infants. Sibling correlation was not accounted for in the 108-infant analysis. **Results:** The study included 108 preterm infants born at 21–25 weeks gestation. Thirty of the infants (27.8%) received a full/booster dose of betamethasone, 31 (28.7%) received an incomplete dose, and 47 (43.5%) did not receive betamethasone. Survival rates in the complete, incomplete, and no-betamethasone groups were 66.7%, 41.9%, and 14.9%, respectively (*p* < 0.001). In the main logistic regression model adjusted for gestational week, sex, and birth weight, compared with the group that did not receive betamethasone, incomplete exposure was associated with a 4.38-fold higher probability of survival, while complete exposure was associated with an 11.95-fold higher probability of survival. Incomplete and complete betamethasone exposure were associated with a lower likelihood of outcomes such as severe intraventricular hemorrhage or death. Complete exposure was also associated with a lower likelihood of the composite outcomes of necrotizing enterocolitis or death and retinopathy of prematurity or death. **Conclusions:** Antenatal betamethasone exposure in the management of periviable infants may offer potential benefits in terms of neonatal survival and morbidity. However, evaluating survival alone as a measure of a successful neonatal outcome is insufficient; serious neonatal morbidities and long-term neurodevelopmental outcomes must also be considered in the clinical decision-making process. To reduce uncertainty in this area, there is a need for studies with larger sample sizes, involving multiple centers, and including long-term follow-up.

## 1. Introduction

The management of infants born at the threshold of viability is one of the most challenging areas of modern neonatology. Although advances in perinatal and neonatal care have pushed the threshold of viability to increasingly earlier gestational ages in extremely preterm infants, survival in these infants still carries significant risks of serious neonatal morbidity and long-term neurodevelopmental problems [1]. For this reason, decisions made during the antenatal period and interventions performed before birth can play a decisive role in the lives of these extremely vulnerable infants.

Antenatal corticosteroid exposure is one of the most important perinatal interventions for improving neonatal outcomes in pregnant women at risk of preterm birth [2]. It is known that betamethasone accelerates fetal lung maturation and reduces neonatal morbidity, particularly respiratory distress syndrome. In addition, it has been shown that the use of antenatal corticosteroids is associated with a reduced risk of intraventricular hemorrhage (IVH), necrotizing enterocolitis (NEC), and neonatal mortality [2,3]. For this reason, antenatal corticosteroid therapy has become one of the standard practices in the management of pregnant women at risk of preterm birth [4]. However, the effect of antenatal corticosteroids on the periviable infants born in the final weeks of pregnancy remains a significant topic of clinical debate.

Statements from the Eunice Kennedy Shriver National Institute of Child Health and Human Development (NICHD), the American Academy of Pediatrics, the American College of Obstetricians and Gynecologists, and the Society for Maternal-Fetal Medicine recommend the use of antenatal corticosteroids starting at 24 weeks of gestation and suggest considering their use for newborns expected to be born at 23 weeks; however, there is no specific recommendation regarding their use. There are no comments or recommendations regarding its exposure at 22 weeks of gestation [5,6]. Furthermore, the fact that a mere one-day difference in the degree of maturity between a birth at 24 ^0/6^ weeks of gestation and one at 23 ^6/7^ weeks results in a difference in betamethasone exposure could be considered unfair to the 23-week-old infant’s chances of survival. In infants born between 21 and 23 weeks, organ immaturity is severe, and the potential impact of every intervention administered during the antenatal period on survival and morbidity becomes even more significant [7].

Antenatal betamethasone exposure reduces both mortality and significant morbidities—including respiratory distress syndrome, necrotizing enterocolitis, and intracranial hemorrhage—in preterm infants [2,8].

Given the limited available evidence regarding the effect of antenatal betamethasone exposure on survival and serious neonatal morbidities, particularly in infants at the limit of viability, and the variability of factors affecting mortality and morbidity in this population, it may be necessary to reevaluate the gestational weeks at which antenatal corticosteroids are administered.

From a neonatologist’s perspective, antenatal betamethasone is not merely a pharmacological treatment administered before birth. When considering the first minutes, hours, and days after birth for a neonate expected to be born on the brink of life, a small intervention performed before birth has the potential to alter the clinical course. In the eyes of a neonatologist, any intervention that could affect the survival of a neonate on the brink of life is not merely a statistical outcome, but also a critical clinical milestone on the line between life and death.

For this reason, determining the potential effects of antenatal betamethasone on survival and neonatal morbidity in neonates at the threshold of life is important from both obstetric and neonatal care perspectives. This information can serve as a guide in antenatal counseling for families, in planning active neonatal care decisions, and in clinical decisions made by the perinatal team before delivery.

This study aimed to evaluate the relationship between antenatal betamethasone exposure and neonatal outcomes in newborns born at the limit of viability. In particular, the study aimed to compare survival rates and major neonatal morbidities between newborns who received antenatal betamethasone and those who did not. Thus, the study sought to determine whether antenatal betamethasone exposure was associated with differences in neonatal outcomes among infants born at the threshold of viability.

## 2. Materials and Methods

### 2.1. Study Design and Population

This study was conducted following approval from the Mersin City and Education Hospital Ethics Committee for Non-Interventional Clinical Research and Publications (20 May 2026/266). This study was conducted at Mersin City and Education Hospital between 1 January 2021 and 1 January 2026; it included newborns born between 21 ^0/7^ and 25 ^6/7^ weeks of gestation who underwent resuscitation in the delivery room and were admitted to the neonatal intensive care unit. Infants with major congenital anomalies or chromosomal abnormalities, those who did not receive resuscitation in the delivery room due to extreme immaturity or a major congenital anomaly, and those with incomplete medical records were excluded from the study.

Multiple pregnancies were eligible. The cohort comprised 108 infants, including 20 recorded as twin births and 88 singleton infants. Pregnancies exposed to corticosteroids that subsequently delivered beyond 25 ^6/7^ weeks were outside this neonatal cohort.

### 2.2. Data Collection

Demographic, perinatal, clinical, and outcome data were obtained from hospital electronic medical records and the neonatal intensive care unit (NICU) database. Maternal and neonatal variables included maternal age, mode of delivery, multiple pregnancy, pregnancy complications, gestational age, birth weight, sex, Apgar scores at 1 and 5 min, and the need for resuscitation at birth.

With regard to antenatal corticosteroid exposure, betamethasone exposure, the number of doses received, and the time interval between the last dose and delivery were recorded. Antenatal corticosteroid exposure was categorized as a full course, an incomplete course, or no exposure, based on available medical records.

Clinical characteristics of neonates included the need for respiratory support, surfactant exposure, mechanical ventilation, the duration of invasive and non-invasive respiratory support, and oxygen requirements, as well as the timing of the first enteral feeding and the time to transition to full enteral feeding.

In addition, antenatal magnesium therapy, the number of days the infant spent in the hospital, and mortality and morbidity outcomes were recorded. Gestational age was determined using obstetric measurements (standard obstetric parameters, last menstrual period, and ultrasound) and based on the gestational weeks defined by the Modified Ballard examination adapted for Turkish children [9].

Antenatal corticosteroid exposure included infants exposed to partial, complete, or repeated courses of antenatal corticosteroids [10].

Incomplete antenatal steroid therapy is defined as a single dose administered within 24 h before delivery, while complete therapy is defined as two intramuscular doses of betamethasone administered 24 h apart. A repeat or booster dose is defined as the exposure of a single 12 mg dose of betamethasone to mothers who have not yet given birth 7 days after the initial course of corticosteroids [11].

Dexamethasone is not used for antenatal corticosteroid treatment at our hospital.

### 2.3. Neonatal Outcomes

The primary outcome is neonatal mortality, while secondary outcomes include length of hospital stay, duration of mechanical ventilation, duration of respiratory support, and the occurrence of significant neonatal morbidities. Morbidities such as bronchopulmonary dysplasia (BPD), intraventricular hemorrhage (IVH), necrotizing enterocolitis (NEC), retinopathy of prematurity (ROP), late-onset sepsis, and the presence of a patent ductus arteriosus (PDA) were recorded. Definitions were established in accordance with relevant guidelines and the literature [12,13,14,15,16].

Composite outcomes were defined a priori. The composite outcome of grade ≥ 3 intraventricular hemorrhage or death was defined as the occurrence of either grade ≥ 3 IVH or death before hospital discharge. Similarly, the composite outcomes of necrotizing enterocolitis or death and retinopathy of prematurity or death were defined as the occurrence of the respective morbidity or death before hospital discharge. Survival without serious morbidity was defined as survival to hospital discharge without any of the serious neonatal morbidities predefined in the study protocol.

Length of hospital stay and discharge status were recorded. Survival was defined as remaining alive until discharge from the hospital.

### 2.4. Ethical Considerations

The study was conducted in accordance with the principles of the Declaration of Helsinki. Ethical approval was obtained from the Mersin City and Education Hospital Ethics Committee/Institutional Review Board on 20 May 2026/266, with 266 approval number. As this was a retrospective study based on existing medical records, the requirement for informed consent was waived by the ethics committee.

### 2.5. Statistical Analysis

Descriptive statistics were presented as the median (IQR), count, and percentage. For group comparisons, the Kruskal–Wallis or Mann–Whitney U test was used for continuous variables, while the Pearson chi-square test, chi-square test adjusted for continuity, or Fisher’s exact test was used to compare categorical variables based on cell frequencies. Results regarding the betamethasone-to-delivery interval were evaluated descriptively due to the limited number of cases in the subgroups. The independent association between betamethasone exposure and clinical outcomes was examined using multivariate logistic regression analysis. The main models were adjusted for gestational week, sex, and birth weight; birth weight was modeled in 100 g increments. Models incorporating preterm membrane rupture and magnesium sulfate were evaluated as sensitivity analyses. Results were reported as adjusted odds ratios (Adj. OR), 95% confidence intervals (CI), and *p*-values. The interaction between betamethasone exposure and gestational age group was examined using a separate logistic regression model with mortality as the dependent variable. In-hospital survival was assessed using the Kaplan–Meier method, and groups were compared using the log-rank test. An event was defined as death, and censoring was defined as discharge or transfer. A *p*-value of <0.05 was considered statistically significant.

## 3. Results

A total of 108 infants born between 21 and 25 weeks of gestation were included in the study. Thirty of the infants (27.8%) received a complete/booster dose of betamethasone, 31 (28.7%) received an incomplete dose, and 47 (43.5%) did not receive betamethasone. No significant differences were found between the groups in terms of sex, gestational age, birth weight, mode of delivery, or multiple pregnancy (*p* > 0.05). Maternal age, 5 and 10 min Apgar scores, early membrane rupture (EMR), magnesium sulfate use, and the frequency of prenatal pathology showed significant differences between the groups (*p* < 0.05) (Table 1).

Survival rates in the complete, incomplete, and no-betamethasone groups were 66.7%, 41.9%, and 14.9%, respectively (*p* < 0.001). Survival rates without serious morbidity were 16.7%, 16.1%, and 0.0%, respectively (*p* = 0.014). The composite outcome of severe IVH or death occurred in 40% of the complete group, 64.5% of the incomplete group, and 87.2% of the group that did not receive betamethasone (*p* < 0.001). While there were no significant differences between the groups in the incidence of severe IVH or any IVH (*p* = 0.158 and *p* = 0.378, respectively), the incidence rates of NEC, NEC or death, ROP, and ROP or death showed significant differences (*p* < 0.05) (Table 2).

In the main logistic regression model adjusted for gestational age, sex, and birth weight, compared with the group that did not receive betamethasone, incomplete exposure was associated with higher odds of survival (adjusted OR = 4.38; 95% CI: 1.38–13.86; *p* = 0.012), while complete exposure was associated with substantially higher odds of survival. (Adj. OR = 11.95; 95% CI: 3.69–38.66; *p* < 0.001). Incomplete exposure (Adj. OR = 0.27; 95% CI: 0.08–0.87; *p* = 0.028) and complete betamethasone exposure (Adj. OR = 0.10; 95% CI: 0.03–0.33; *p* < 0.001) were associated with a lower likelihood of the composite outcome of severe IVH or death. Complete exposure was also associated with a lower likelihood of the composite outcomes of NEC or death (Adj. OR = 0.25; 95% CI: 0.07–0.87; *p* = 0.030) and ROP or death (Adj. OR = 0.06; 95% CI: 0.01–0.60; *p* = 0.016). In sensitivity analyses where EMR and magnesium sulfate were added to the model, the direction of the association generally remained unchanged, although some confidence intervals widened (Table 3).

In a comparison by gestational age group, the birth weight of infants at 24–25 weeks was found to be higher than that of infants at 21–23 weeks (680 [600–765] vs. 510 [480–570] g; *p* < 0.001). The cesarean delivery rate was higher in the 24–25-week group (*p* = 0.018), and the length of hospital stay was longer (*p* = 0.030). No significant differences were found between groups in the rate of betamethasone exposure, survival, mortality, survival without serious morbidity, severe IVH, NEC, ROP, and duration of mechanical ventilation (*p* > 0.05) (Table 4).

Survival was additionally presented by individual completed gestational week (Table 5). At 21 weeks, none of the three infants survived. At 22 weeks, 7/12 exposed and 0/13 unexposed infants survived (*p* = 0.002). These small, exploratory comparisons do not establish treatment-effect differences between gestational weeks (Table 5).

In the subgroup of infants aged 21–23 weeks, survival was 48.4% in those who received betamethasone and 7.7% in those who did not (*p* = 0.001). The composite outcome of severe IVH or death was 54.8% in those who received betamethasone and 92.3% in those who did not (*p* = 0.003). While there were no significant differences between the groups in survival without morbidity or the incidence of severe IVH (*p* = 0.242 and *p* = 0.228, respectively), the incidence of NEC and ROP was higher in the group receiving betamethasone (*p* = 0.029 and *p* = 0.008, respectively). Mechanical ventilation and hospital stay durations were longer in the betamethasone group (*p* < 0.001 for both). Differences in isolated NEC and ROP outcomes and duration of supportive care should be interpreted with consideration of survival bias due to early mortality (Table 6).

When the post-betamethasone birth interval was examined for the 61 infants who received betamethasone, the highest observed survival rate was found in the 12–24 h group (81.8%), while the lowest survival rate was found in the 6–12 h group (25.0%). The survival rate was 43.3% in the 0–6 h group, 71.4% in the 24–72 h group, and 55.6% among those who received betamethasone within the past week (Table 7).

**Figure 1:**
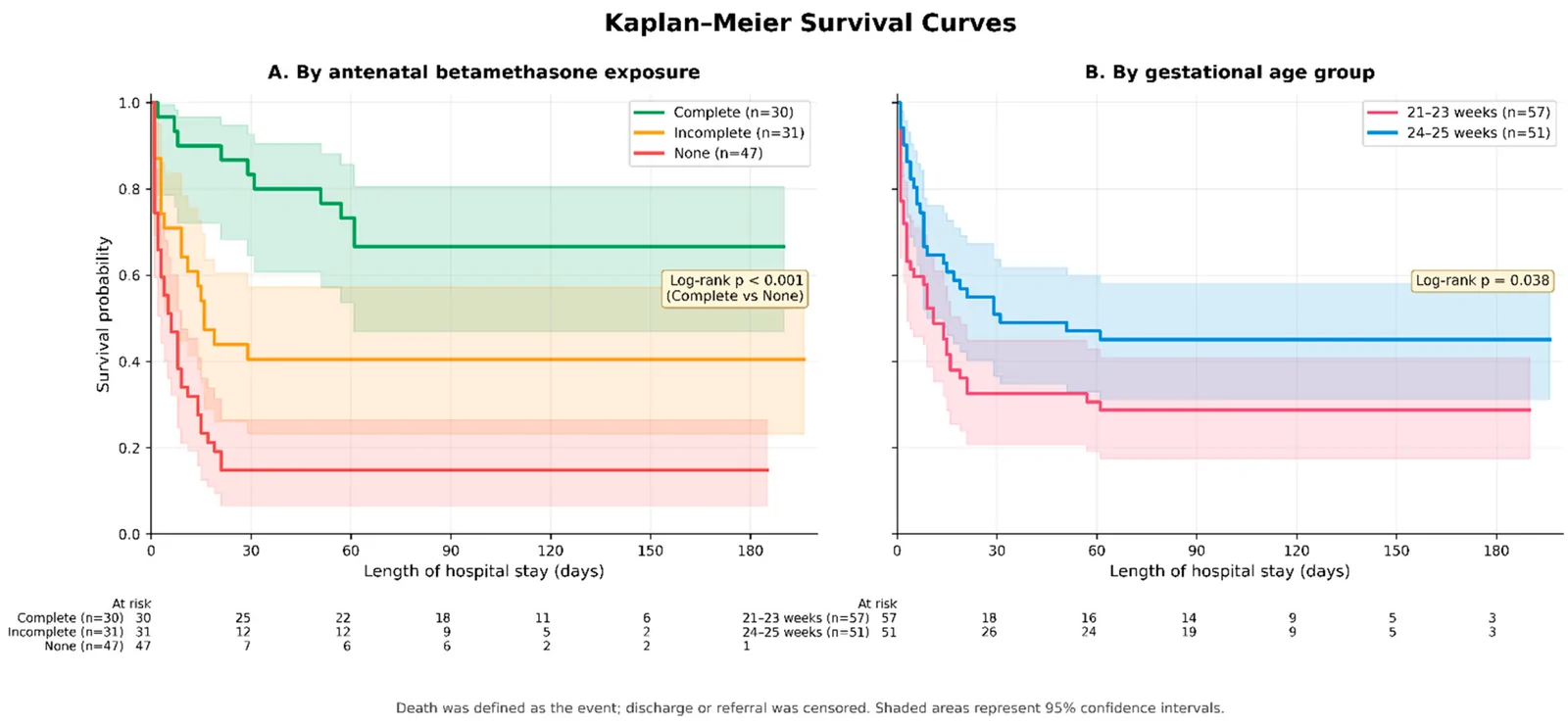
Kaplan–Meier survival curves by antenatal betamethasone exposure and gestational age group. Panel (**A**) shows the complete, incomplete, and betamethasone-naive groups; Panel (**B**) shows the groups at 21–23 and 24–25 weeks of gestation. Death, event, discharge, or transfer were considered censoring events. The shaded areas indicate the 95% confidence intervals.

No statistically significant interaction between betamethasone exposure and gestational age group was found (OR = 1.58; 95% CI: 0.19–13.11; *p* = 0.671). However, due to the wide confidence interval, it could not be concluded that the effect of betamethasone was equivalent across gestational age groups. In the Kaplan–Meier analysis, a significant difference in in-hospital survival was found between the complete betamethasone and no-betamethasone groups (*p* < 0.001). Survival curves also showed a significant difference between the 21–23 and 24–25-week gestational age groups (*p* = 0.038) (Figure 1).

## 4. Discussion

One of the most important interventions affecting neonatal outcomes in preterm infants is the exposure of antenatal corticosteroids to the mother before delivery. A randomized controlled trial conducted in 1972 was the first to demonstrate that antenatal corticosteroids reduce respiratory morbidity and mortality in neonates [17].

Following additional randomized studies confirming the efficacy of antenatal corticosteroids, in the mid-1990s the National Institutes of Health Consensus Development Panel concluded that intramuscular betamethasone (two 12 mg doses administered 24 h apart) or dexamethasone (four 6 mg doses administered 12 h apart) is indicated for all pregnant individuals between 24 and 34 weeks of gestation who are at risk of preterm birth [18].

A gestational age of 24 weeks has been chosen as the lower limit because survival before this gestational age is virtually impossible. However, advances in medicine and technology, along with the resulting increase in complication-free survival rates among smaller, more premature neonates, have highlighted the need to reevaluate this threshold [19]. However, while the guidelines state that “antenatal steroids may be administered” at 23 weeks, they do not yet provide clear recommendations for smaller neonates [4].

The aim of this study is to evaluate the effects of antenatal betamethasone treatment—including the number of doses and timing of exposure—on mortality and morbidity in immature neonates born during the periviable weeks over 5 years.

This study is a retrospective cohort study of 10,541 neonates born between 22 and 25 weeks of gestation with a birth weight of 401–1000 g, conducted by the NICHD Neonatal Research Network between 1993 and 2009 [20].

In this study, no significant reduction in mortality or neurodevelopmental disorders was observed in neonates born at 22 weeks’ gestation who received antenatal corticosteroids. At 23 weeks (83.4% vs. 90.5%; aOR 0.58, 95% CI 0.42–0.80), at 24 weeks (68.4% vs. 80.3%; aOR 0.62, 95% CI 0.49–0.78), and at 25 weeks (52.7% vs. 67.9%; aOR 0.61, 95% CI 0.50–0.74), were found to be associated with a reduction in mortality or neurodevelopmental disorders during the 18–22-month period. In addition, they found that neonates born at 22 weeks’ gestation who were exposed to steroids before birth had a lower risk of necrotizing enterocolitis compared to those not exposed to steroids [20].

In our study, survival rates in the complete, incomplete, and no-betamethasone groups were found to be 66.7%, 41.9%, and 14.9%, respectively (*p* < 0.001). Complete/booster and incomplete exposure were associated with higher odds of survival (aOR 11.95 and 4.38, respectively); these estimates are not fold increases in survival probability. Survival rates without serious morbidity were 16.7% in the group that received complete antenatal betamethasone and 0% in the group that did not receive it (*p* = 0.014). This endpoint did not establish survival without all serious morbidities. The incidence of severe IVH or death was 40% in the complete group, 64.5% in the incomplete group, and 87.2% in the group that did not receive betamethasone (*p* < 0.001). Even with a single dose of betamethasone, a lower probability of severe IVH or death was observed. Complete exposure was also found to be associated with a lower likelihood of NEC or death and ROP or death. Our study included infants born at an earlier gestational age, and our results were similar to those of the NICHD Neonatal Research Network. Unlike their findings, we also observed a reduction in the risk of ROP.

Another retrospective cohort study conducted by the NICHD Neonatal Research Network evaluated the effect of antenatal corticosteroids in periviable multiple pregnancies and found that, among 6925 multiple-birth infants born at 22–28 weeks’ gestation, antenatal corticosteroids were associated not only with a reduction in the risk of in-hospital death (aRR 0.87, 95% CI 0.78–0.96), but also with a reduced risk of severe intraventricular hemorrhage or death and necrotizing enterocolitis or death. In our study as well, we observed a reduction in both mortality and the risk of serious morbidity in these periviable infants. Unlike other studies, we also observed a decrease in the incidence of severe ROP in infants whose mothers received betamethasone [21].

Regarding the timing of antenatal corticosteroid exposure, a retrospective cohort study conducted in Sweden on 707 newborns born between 22 and 26 weeks of gestation found that the greatest benefit of antenatal corticosteroids was observed in newborns born 24 h to 7 days after steroid exposure, compared to those administered at shorter or longer intervals [20].

When evaluating the timing of antenatal betamethasone exposure and survival rates, the highest observed survival was seen among infants who received betamethasone 12–24 h before delivery (81.8%); however, this subgroup included only 11 infants. Therefore, these findings should be interpreted as descriptive and do not allow conclusions regarding an optimal interval between antenatal betamethasone administration and delivery.

With regard to short-term morbidity, our study confirms the findings of Chawla et al., who demonstrated that antenatal betamethasone exposure improves survival without causing serious morbidity [22]. However, comparison with Chawla et al. requires caution: our separate morbidity category did not validate survival without serious morbidity. Disease severity and concurrent morbidity ascertainment were incomplete, and early death could prevent ROP examination.

A study published in 2025 evaluated 17 infants born at 21 weeks’ gestation who underwent resuscitation; it was found that of the 7 infants who survived, 3 received a full course of betamethasone, 2 received an incomplete dose, and 2 did not receive any [23].

In our study, the survival rate of infants who received betamethasone between 21 and 23 weeks of gestation was found to be statistically significant. However, limiting mortality in newborns born at such a low gestational age through antenatal corticosteroid exposure alone would not be an appropriate approach. Factors such as antenatal care and maternal morbidities should also be considered as important determinants of mortality and morbidity in these infants.

In our study, many patients in the 21–23-week group could not receive betamethasone or magnesium. This was because the patients presented to the hospital with labor already underway, and the neonates were delivered quickly. The reason betamethasone doses were completed and magnesium infusions were administered in patients with maternal complications or premature rupture of membranes is that these mothers were hospitalized due to their maternal complications and were under close monitoring during the final stages of pregnancy. Moreover, the outcomes for the infants of mothers hospitalized for these reasons are more favorable compared to those in other groups.

Our study has several limitations that should be acknowledged. First, the single-center, retrospective, observational design may limit the generalizability of our findings and precludes causal inference. The decision to administer antenatal corticosteroids was influenced by clinical circumstances and the timing of maternal presentation, and the imbalance in baseline and perinatal characteristics, particularly regarding magnesium sulfate exposure, premature rupture of membranes, prenatal pathology, and Apgar scores, may have introduced selection bias and residual confounding. Although multivariable analyses were adjusted for gestational age, sex, and birth weight, unmeasured or incompletely measured confounders cannot be completely excluded. In addition, infants who died in the delivery room or did not undergo active resuscitation were not included because the study specifically focused on infants receiving active neonatal care and NICU admission, which may further limit generalizability to all infants born at 21–25 + 6 weeks of gestation. Nevertheless, the five-year study period and the role of our institution as a tertiary perinatal referral center allowed inclusion of a relatively substantial and clinically heterogeneous population.

Another limitation is that we did not evaluate pregnancies in which antenatal betamethasone was administered at 21–25 weeks of gestation, but delivery occurred at a later gestational age. Although information on subsequent gestational age at delivery and neonatal morbidity could have provided additional insight into the broader implications of antenatal corticosteroid exposure at the edge of viability, our study was specifically designed to evaluate outcomes among infants actually born at 21–25 weeks of gestation. Therefore, pregnancies continuing beyond this gestational-age range were not included in the present analysis.

Current evidence suggests that antenatal corticosteroids administered during the periviable period after the 21st week of gestation may reduce neonatal mortality and improve survival in appropriately selected pregnant women. However, it should be noted that an increase in survival does not always correspond to a reduction in severe neonatal morbidity or an improvement in long-term neurodevelopmental outcomes. In particular, it is evident that severe complications and long-term sequelae associated with prematurity continue to pose a significant problem, especially among infants born during the periviable period [24].

For this reason, when evaluating antenatal corticosteroid therapy, it is necessary to consider not only short-term survival but also long-term neurodevelopmental outcomes, as well as serious intraventricular hemorrhage, necrotizing enterocolitis, bronchopulmonary dysplasia, sepsis, and other significant neonatal morbidities. On the other hand, the potential adverse effects of antenatal corticosteroid use during the periviable period should not be overlooked. Some studies have reported that corticosteroids administered particularly close to delivery may be associated with certain morbidities, primarily infection [25].

However, since a significant portion of the available data comes from retrospective and observational studies, these associations must be interpreted with caution regarding causality. Therefore, there is a need for prospective, multicenter studies that include long-term follow-up to better elucidate the potential benefits and possible risks of treatment.

## 5. Conclusions

The primary goal in caring for an infant born at the periviable stage should extend beyond mere survival. Rather, the ultimate objective should be to achieve the highest possible quality of life by minimizing long-term neurodevelopmental impairment, significant functional disabilities, and lifelong healthcare needs. In the periviable population, the fundamental question is not simply whether an infant survives, but rather how that infant survives. Ultimately, the goal should be to ensure that survival translates into the best possible quality of life. Antenatal betamethasone exposure in neonates born at 21–25 weeks of gestation was associated with higher survival and a lower incidence of severe neonatal morbidity. These findings suggest that even a single dose of antenatal corticosteroids may offer clinically meaningful benefits when delivery is imminent and insufficient time is available to complete the standard corticosteroid course. From a neonatologist’s perspective, therefore, antenatal betamethasone should not be regarded solely as an intervention aimed at improving survival among infants born at 21–25 weeks of gestation, but rather as a potentially important antenatal intervention that may contribute to improved short-term neonatal outcomes. However, these findings should be interpreted cautiously, given the potential for residual confounding, selection into active care, incomplete ascertainment of morbidity, and unaccounted sibling correlation. Importantly, the observational nature of the study precludes causal inference and does not establish a general reduction in serious morbidity, long-term neurodevelopmental benefit, or benefit specifically at 21 weeks of gestation.

Current evidence supports a potential survival benefit of antenatal corticosteroid exposure in women at risk of periviable birth; however, uncertainty remains regarding its impact on long-term outcomes. Ultimately, every decision made during the periviable period should extend beyond the question of “Can we save the baby?” also to consider the more fundamental question of “What kind of life can we help this baby achieve?”

In conclusion, further large-scale, multicenter studies incorporating long-term neurodevelopmental outcomes are needed to determine the optimal use of antenatal corticosteroids in periviable pregnancies. Such evidence will provide important guidance for neonatologists in assessing prognosis and informing clinical decision-making for these vulnerable infants.

## Figures and Tables

**Table 1:** Maternal and neonatal characteristics by betamethasone exposure.

	Complete (*n* = 30)	Incomplete (*n* = 31)	None (*n* = 47)	*p*
**Sex (Male), ** ***n*** ** (%)**	11 (36.7)	13 (41.9)	26 (55.3)	0.235
**Maternal Age (years)**	29.5 (25–33)	31 (26–36)	25 (21.5–30)	**0.001**
**Gravity**	2 (2–3.8)	2 (1–5)	2 (1–3)	0.329
**Parity**	2 (1–3.8)	2 (1–4)	2 (1–3)	0.679
**Gestational age (week)**	24 (23–24)	23 (23–24)	23 (22–24)	0.512
**21–23 weeks, ** ***n*** ** (%)**	13 (43.3)	18 (58.1)	26 (55.3)	0.462
**Birth weight (g)**	600 (540–660)	540 (505–685)	580 (500–660)	0.573
**Cesarean delivery, ** ***n*** ** (%)**	20 (66.7)	23 (74.2)	26 (55.3)	0.221
**Multiple pregnancy, ** ***n*** ** (%)**	3 (10)	7 (22.6)	10 (21.3)	0.364
**Early membrane rupture, ** ***n*** ** (%)**	16 (53.3)	4 (12.9)	0 (0)	**<0.001**
**Magnesium sulfate, ** ***n*** ** (%)**	29 (96.7)	10 (32.3)	2 (4.3)	**<0.001**
**Prenatal problem, ** ***n*** ** (%)**	25 (83.3)	26 (83.9)	23 (48.9)	**0.001**
**Apgar 5. min**	5 (3.2–5)	3 (3–4)	3 (2–5)	**0.004**
**Apgar 10. min**	6 (5–6)	5 (5–6)	5 (5–6)	**0.045**
**CPR: PBV/IT, ** ***n*** ** (%)**	24 (80)	19 (61.3)	28 (59.6)	0.056
**CPR: massage/drug, ** ***n*** ** (%)**	2 (6.7)	11 (35.5)	15 (31.9)	
**Surfactant doses**	2 (2–3)	2 (1–2)	2 (1–3)	0.063

CPR: Cardiopulmonary resuscitation, PBV: Positive pressure ventilation, IT: endotracheal intubation, min: minutes. Data presented as median (IQR) or *n* (%).

**Table 2:** Survival and morbidity by betamethasone treatment.

Conclusion	Complete	Incomplete	None	*p*
**Survival**	20/30 (66.7)	13/31 (41.9)	7/47 (14.9)	**<0.001**
**Survival without serious morbidity**	5/30 (16.7)	5/31 (16.1)	0/47 (0)	**0.014**
**Mortality**	10/30 (33.3)	18/31 (58.1)	40/47 (85.1)	**<0.001**
**Severe IVH**	3/30 (10)	3/31 (9.7)	11/47 (23.4)	0.158
**Grade 3 or higher IVH or death**	12/30 (40)	20/31 (64.5)	41/47 (87.2)	**<0.001**
**Grade 1–2 IVH**	18/30 (60)	22/31 (71)	26/47 (55.3)	0.378
**NEC**	15/30 (50)	14/31 (45.2)	10/47 (21.3)	**0.017**
**NEC or death**	20/30 (66.7)	23/31 (74.2)	42/47 (89.4)	**0.046**
**ROP**	13/30 (43.3)	7/31 (22.6)	6/47 (12.8)	**0.009**
**ROP or death**	23/30 (76.7)	25/31 (80.6)	46/47 (97.9)	**0.012**

NEC: Necrotizing enterocolitis; ROP: retinopathy of prematurity; IVH: Intraventricular hemorrhage.

**Table 3:** Multivariate logistic regression analysis of the relationship between betamethasone treatment and clinical outcomes.

Conclusion	Comparison	Base Model Adj. OR (95% CI)	*p*	Sensitivity Model Adj. OR (95% CI)	*p*
**Survival**	Incomplete vs. none	4.38 (1.38–13.86)	**0.012**	5.50 (1.63–18.60)	**0.006**
	Complete vs. none	11.95 (3.69–38.66)	**<0.001**	30.11 (4.21–215.53)	**0.001**
**Severe IVH or death**	Incomplete vs. none	0.27 (0.08–0.87)	**0.028**	0.23 (0.07–0.77)	**0.018**
	Complete vs. none	0.10 (0.03–0.33)	**<0.001**	0.05 (0.01–0.33)	**0.002**
**NEC or death †**	Incomplete vs. none	0.33 (0.09–1.22)	0.096	0.27 (0.07–1.05)	0.059
	Complete vs. none	0.25 (0.07–0.87)	**0.030**	0.10 (0.01–0.89)	**0.039**
ROP or death *	Incomplete vs. none	0.08 (0.01–0.78)	**0.030**	0.06 (0.01–0.56)	**0.014**
	Complete vs. none	0.06 (0.01–0.60)	**0.016**	0.01 (<0.01–0.20)	**0.005**

NEC: Necrotizing enterocolitis; ROP: retinopathy of prematurity; IVH: Intraventricular hemorrhage. † Main model: gestational age, sex, and birth weight. Sensitivity model: the main model plus EMR and magnesium sulfate. * Due to the small number of adverse events, the results should be considered exploratory. Adj. OR: adjusted odds ratio; CI: confidence interval.

**Table 4:** Characteristics and outcomes by gestational age group.

	21–23 Week (*n* = 57)	24–25 Week (*n* = 51)	*p*
**Maternal Age (years)**	29 (23–34)	27 (24–31.5)	0.450
**Birth weight (g)**	510 (480–570)	680 (600–765)	**<0.001**
**Apgar 5. min**	4 (2–5)	4 (3–5)	0.310
**Cesarean, ** ***n*** ** (%)**	30 (52.6)	39 (76.5)	**0.018**
**Early membrane rupture, ** ***n*** ** (%)**	10 (17.5)	10 (19.6)	0.978
**Magnesium sulfate, ** ***n*** ** (%)**	20 (35.1)	21 (41.2)	0.651
**Betamethasone, ** ***n*** ** (%)**	31 (54.4)	30 (58.8)	0.787
**Survival, ** ***n*** ** (%)**	17 (29.8)	23 (45.1)	0.149
**Mortality, ** ***n*** ** (%)**	40 (70.2)	28 (54.9)	0.149
**Survival without morbidity, ** ***n*** ** (%)**	3 (5.3)	7 (13.7)	0.237
**Grade 3 and higher IVH, ** ***n*** ** (%)**	7 (12.3)	10 (19.6)	0.436
**NEC, ** ***n*** ** (%)**	23 (40.4)	16 (31.4)	0.442
**ROP, ** ***n*** ** (%)**	11 (19.3)	15 (29.4)	0.316
**MV duration (days)**	11 (2–35)	21 (8–38)	0.076
**Duration of hospitalization (days)**	11 (2–81)	31 (8–104)	**0.030**

NEC: Necrotizing enterocolitis; ROP: retinopathy of prematurity; IVH: Intraventricular hemorrhage; MV: mechanical ventilation.

**Table 5:** Survival by completed gestational week.

Gestational Week	Total (*n*)	Exposed Survival *n*/N (%)	Unexposed Survival *n*/N (%)	*p*
21	3	0/1 (0.0)	0/2 (0.0)	—
22	25	7/12 (58.3)	0/13 (0.0)	**0.002**
23	29	8/18 (44.4)	2/11 (18.2)	0.234
24	31	10/18 (55.6)	2/13 (15.4)	0.032
25	20	8/12 (66.7)	3/8 (37.5)	0.362

**Table 6:** Outcomes according to betamethasone exposure in 21–23 week infants.

Conclusion	Betamethasone (*n* = 31)	No Betamethasone (*n* = 26)	*p*
**Survival**	15/31 (48.4)	2/26 (7.7)	**0.001**
**Mortality**	16/31 (51.6)	24/26 (92.3)	**0.001**
**Survival without morbidity**	3/31 (9.7)	0/26 (0)	0.242
**Severe IVH**	2/31 (6.5)	5/26 (19.2)	0.228
**Severe IVH or death**	17/31 (54.8)	24/26 (92.3)	**0.003**
**NEC**	17/31 (54.8)	6/26 (23.1)	**0.029**
**ROP**	10/31 (32.3)	1/26 (3.8)	**0.008**
**MV duration (days)**	21 (8–60)	3 (1–10)	**<0.001**
**Duration of hospitalization (days)**	57 (8–125)	3 (1–10)	**<0.001**

NEC: Necrotizing enterocolitis; ROP: retinopathy of prematurity; IVH: Intraventricular hemorrhage; MV: mechanical ventilation.

**Table 7:** Outcomes by betamethasone–birth interval.

Time	*n*	Survival *n* (%)	Mortality *n* (%)	Severe IVH *n* (%)	NEC *n* (%)	ROP *n* (%)
0–6 h	30	13 (43.3)	17 (56.7)	3 (10.0)	13 (43.3)	7 (23.3)
6–12 h	4	1 (25.0)	3 (75.0)	0 (0.0)	2 (50.0)	0 (0.0)
12–24 h	11	9 (81.8)	2 (18.2)	1 (9.1)	6 (54.5)	6 (54.5)
24–72 h	7	5 (71.4)	2 (28.6)	0 (0.0)	5 (71.4)	4 (57.1)
In the past week	9	5 (55.6)	4 (44.4)	2 (22.2)	3 (33.3)	3 (33.3)

NEC: Necrotizing enterocolitis; ROP: retinopathy of prematurity; IVH: Intraventricular hemorrhage.

## Data Availability

The data presented in this study are available on request from the corresponding author. The data are not publicly available due to privacy and ethical restrictions, as they contain sensitive personal and health-related information.
